# Structure, conservation and health implications of urban wild meat value chains: A case study of Lagos, Nigeria

**DOI:** 10.1016/j.onehlt.2025.100992

**Published:** 2025-02-14

**Authors:** Samuel N. Akpan, Pim van Hooft, Anise N. Happi, Ralph Buij, Frank van Langevelde, Elizabeth A.J. Cook, James M. Hassell, Dawn M. Zimmerman, Sherrill P. Masudi, Christian T. Happi, Lian F. Thomas

**Affiliations:** a Wageningen University & Research, P.O. Box 9101, 6700, HB, Wageningen, the Netherlands; b Institute of Genomics and Global Health, Redeemers University, PMB 230 Ede, Nigeria; c Wageningen Environmental Research, P.O. Box 47, 6700, AA, Wageningen, the Netherlands; d International Livestock Research Institute, PO Box 30709-00100y, GPO Nairobi, Kenya; e Smithsonian Conservation Biology Institute, 3001 Connecticut Ave NW, Washington, DC 20013, USA; f Department of Epidemiology of Microbial Disease, Yale School of Public Health, New Haven, CT, USA; g Smithsonian Institution–National Museum of Natural History, Department of Entomology, 10th St. & Constitution Ave. NW, Washington, DC 20560, USA; h Harvard T.H. Chan School of Public Health, 677 Huntington Ave, Boston, MA 02115, USA; i Royal (Dick) School of Veterinary Studies, University of Edinburgh, Easter Bush Campus, Midlothian EH25 9RG, United Kingdom

**Keywords:** Wild meat trade, Bushmeat, Zoonotic spillover, Public health, Wildlife species, Gender, Conservation, Value chains

## Abstract

Urban wild meat value chains represent a vital source of protein and livelihood intertwined with culture and complex market systems. Against the backdrop of escalating zoonotic disease concerns linked to wild meat, this research aimed to unravel the structure, governance, species composition and actor characteristics of the wild meat value chain in Lagos, Africa's most populated city. We employed a cross-sectional study design incorporating qualitative and quantitative approaches. Interviews (*n* = 22) were conducted and structured questionnaires were administered to participants (*n* = 257) across 15 sites in Lagos. Participants' activities were also observed, and field notes were taken. Descriptive statistics and inductive thematic approach were used for data analysis. Findings revealed five main value chain nodes: hunter, processor, wholesale, retail and consumer; and five major geographical areas from which wild meat flowed into the city. Governance structures showed a blend of informal and formal mechanisms sustained through trust, cultural beliefs and informal regulations. The main motivation was income (88.1 %), and period of optimum activity was at night times (53 %) during the dry season (≥ 62 %). Women (62.2 %) dominated the value chain, prevalent at the wholesaler (61.7 %), processor (89.7 %) and retailer (83 %) nodes. Hunters (40 %) were able to supply wild meat directly to consumers, and the large-scale retailers (26 %) were responsible for exportation of wild meat. A total of 35 species were traded in the value chain, and the most commonly traded species were: grasscutters, duikers, porcupines, and giant-pouched rats (≥90.7 % participants). Pottos, buffalos, tortoises and house snakes were the least traded (≤ 7.0 % participants). This study provides an understanding of Lagos wildlife trade from a value chain perspective, paving the way for interventions to address wildlife conservation challenges and spillover risks. Species traded in the value chain have been globally reported as reservoirs of zoonotic pathogens, representing a source of zoonotic spillover risks to actors. Also, the ability of hunters to supply wild meat directly to consumers signals an increase in the speed of zoonotic pathogen spread, and portends a greater risk for public health. Formal governance should be integrated into the value chain to aid effective monitoring and regulation. Conservation and public health interventions should be node-specific and gender-sensitive, targeting the dry seasons when actors' activities peak, and risks of human-wild meat contact are greater. There is need for the re-assessment of the conservation statuses of West Africa's wildlife species to reflect current realities foisted by wild meat trade.

## Highlights

• Wild meat activities peaked in the dry season, signifying a a period with greater risks of zoonotic spillover.
• The direct supply of wild meat from hunters to homes and other end users may increase speed of potential zoonoses transmission.
• "Social norms" and "income" emerged as the main drivers of wild meat trade in Lagos.
• Species abundance, ease of capture and culture play vital roles in the availability of certain species in wild meat markets.
• Female actors operated a more formal governance structure which could be monitored and regulated.

## 1 Introduction

Wild meat, also referred to as “bushmeat”, holds a prominent position in the diets of many communities, particularly in tropical and subtropical regions where it contributes significantly to nutritional diversity and food security [1,2]. The consumption and trade of wild meat form intricate value chains influenced by a myriad of factors including cultural traditions, economic drivers, and security concerns, particularly within urban environments [3,4]. However, the global implications of this practice are substantial, with the widespread consumption of wild meat contributing to large scale defaunation and biodiversity loss [5].

In sub-Saharan Africa, wild meat is a highly prized non-timber forest product [6] with their consumption deeply ingrained in the dietary habits, livelihood strategies, and cultural customs of many societies [7,8]. It serves not only as a significant source of protein but also as a vital component of local economies [9]. The increasing demand and trade for wild meat within urban centers has been fueled by population increase due to urbanization [10]. The desire of people in urban areas to eat wild meat is a crucial factor in promoting the over-exploitation of wildlife in many regions of the world [11]. Importantly, the sustainability and ethical implications of wild meat exploitation raise profound concerns, which in addition to wildlife conservation especially also concerns public health [7,8]. Despite previous health crises linked to wild meat, such as Ebola, the trade in wild meat continues, highlighting the entrenched nature of cultural practices and the challenges in shifting consumer behaviors [12]. The informality and regulation hurdles in unofficial urban wild meat market systems impede regulatory efforts; resulting in problems for human health, hygiene, and traceability. The lack of appropriate legislation makes it more difficult to ensure safe wild meat consumption and trade practices [13]. These further exacerbate the risk of zoonotic disease outbreaks and threatens already vulnerable wildlife populations. According to the World Health Organization [14], 75 % of emerging infectious diseases in the last decade has been of zoonotic origin. Trade in wild meat has been associated with outbreaks of SARS, HIV, and Ebola [15]. Also, the ecological effects of the urban wild meat trade as examined by Lindsey et al. [16] include adverse impacts on biodiversity, with unsustainable hunting methods contributing to species extinction. To effectively curtail emerging disease outbreaks linked to wild meat consumption and implement sustainable management practices, it is imperative to comprehend the trends, causes, and effects of wildlife trade in urban environments.

In Nigeria, wild meat consumption and trade are deeply entrenched cultural practices, providing essential protein sources and livelihood opportunities for local populations [17,18]. The Lagos landscape, characterized by rapid urbanization and a diverse consumer demography, shapes the dynamics of the wild meat value chain in unique ways. Despite ongoing efforts to regulate the trade, the wild meat market in Lagos operates largely uncontrolled, presenting significant challenges for wildlife conservation and public health management [19,20]. This lack of regulation is particularly concerning given the persistent demand for wild meat in many countries of West and Central Africa, where it remains a vital protein source and revenue generator [17,21].

Urban centers are transport and market hubs that form hotspots of import/export of resources such as wildlife and/or wild meat, thus promoting the rapid and wide-scale spread of reservoir and vectors, and creating conducive environments for viral recombination and bacterial plasmid exchanges [22,23]. While studies have been conducted on this subject in some African countries, little is known about the structure, governance, health and ecological aspects of wild meat trade in Lagos, Africa's most populated city. The status of Lagos as the international commercial hub of the West African region could make it an important gateway for spread of pathogens to other parts of the globe. World urban population has been estimated to rise rapidly between years 2015 and 2025, such that 62 % of the global human population will live in the urban areas [24]. It is therefore pertinent to investigate the global implications of wild meat trade in rapidly urbanizing cities.

This study addresses the following research questions: (i) what are the structural elements of the wild meat value chain in Lagos? (ii) what is the governance structure, and who are the actors involved? (iii) which species are traded? (iv) what are the implications of the wild meat value chain on public health and conservation? Through a comprehensive examination of the value chain dynamics, species and key actors involved, this research contributes to a deeper understanding of the complexities surrounding how wild meat is extracted, traded and regulated in urban city settings using Lagos as a case study.

## 2 Materials and methods

### 2.1 Ethical approvals

This study received approval from the National Health Research Ethics Committee (NHREC) of Nigeria (Approval number: NHREC/01/01/2007–07 /12/2022). We received verbal consents from all the participants, after a formal introduction and description of the study, with the use of an informed consent sheet (**Supplementary File 1**). This process was witnessed by the lead author.

### 2.2 Study area

This study was conducted in Lagos, Africa's most populated city center. Regarded as the commercial nerve center of West Africa, Lagos boasts a metropolitan human population of 15.9 million people [25] as at 2023. Due to its geographical location, it is easily accessible by land, air and water. The study's target population included key stakeholders in the urban wild meat value chain, such as hunters, traders, and consumers. The study utilized purposive sampling [26] to select participants based on their relevance to the study. These were retailers (100), hunters (55), processors (68), and wholesalers (34).

### 2.3 Data collection

Qualitative data collection methods, including in-depth key informant interviews (KII) and participant observation were combined with quantitative structured surveys to gain a comprehensive understanding of actors' activities and market dynamics.

#### 2.3.1 Identification of value chain channels

A total of twenty-four (24) points of wild meat value chain activities were identified and visited in 15 towns in the Lagos metropolis: Epe, Igbe-lara, Imota, Itoikin, Sangotedo, Ikoyi, Ajah, Surulere, Ebute-Metta, Ikeja, Ikorodu, Ifako-Ijaiye, Okota, Badagry and Obalende. These comprised hunter aggregation points, markets, shops, road-side sales outlets, restaurants and drinking bars. This was carried out over a four-month period in 2023 (September–December), cutting across the rainy (September–October) and the dry seasons (November–December).

#### 2.3.2 Questionnaire administration

Semi-structured questionnaires (**Supplementary File 2**) were used to collect quantitative data on actors' demography, species of wild meat traded, market dynamics, and the motivations driving the actors. A local language interpreter was used, as well as community/market leads. The local interpreter read out the questions to the participants and filled out their answers into the questionnaires. However, this approach had its potential bias, as reading out and interpretation of questions to the participants in their local language may unknowingly drive certain responses.

#### 2.3.3 Key informant interviews

Key informants were selected by identifying market heads and leaders among the hunters and wild meat traders, to gather insights into their roles, practices and modes of operation. Based on the criteria of willingness and availability to participate, twenty-two (22) key informants agreed to participate. Using a semi-structured interview guide (**Supplementary File 3**), and with the help of a local language interpreter, we asked questions about their roles in the value chain, governance structures, and their practices. We also asked follow-up questions to confirm and screen responses, gather different perspectives and receive new information. When new pieces of information were received, we probed further to gain clearer insights on that specific information. Each interview lasted between 25 and 40 min, and all responses were audio-recorded.

#### 2.3.4 Capturing of geographic data

We used a Garmin Etrex 10 GPS Navigator tool to record coordinates of the respective value chain nodes visited.

#### 2.3.5 Participants' observation

Participants' observations were utilized to gain a first-hand understanding of the traded species, practices and other dynamics within the urban wild-meat value chain. This was at the hunter and wholesaler nodes in Epe, Imota, Itoikin, Badagry and Igbe-Lara areas, where species were yet to be processed and hence phenotypic identification was possible. Field notes were written, and photographs were also taken to support species identification.

### 2.4 Data analysis

Freshly killed wildlife species seen during participants' observation process were phenotypically identified by a wildlife expert on the research team. Content analysis was used to analyze other observations which were written as field notes. For qualitative data from interviews, the thematic analytical method was used to identify recurring themes, patterns, and variations in the narratives of different actors (**Supplementary File 4**). The processes mentioned by Braun and Clarke [27], which include data transcription and organization, familiarization, coding, theme production, review, theme refinement, and data interpretation and reporting, were applied. First, we transcribed our interviews and read them repeatedly to familiarize ourselves with the raw data. Adopting an inductive approach, we sought for themes to emerge from the transcript. We manually wrote out short texts from the transcript on sticky notes. We read through the texts on each sticky note, and repeated the process, this time assigning initial codes identified from the texts to colors (eg. green: culture). We repeated this process until each text was written to a color-coded sticky note relevant for each. We read the transcripts again to ascertain that we did not miss any text or information. Carefully reading through the initial codes, we identified related groups, and categorized them into themes. We then sorted the color-coded notes and placed them together based on related themes. We defined the themes, and repeatedly went through the codes again to ensure that each one was rightly placed under a befitting theme, or needed a new theme. We performed re-groupings as necessary, and inputted the analyzed data on to a Microsoft Excel sheet for ease of writing. Quantitative data was analyzed using descriptive statistical and inferential analysis using Microsoft Excel (**Supplementary File 5**). Geographic positioning system data was analyzed using the ArcGIS software.

## 3 Results

### 3.1 Structural elements of the value chain

Five main nodes were identified in the Lagos wild meat value chain. These were: Hunter, Wholesaler, Processor, Retailer, and Consumer. There were two categories of hunters: the “commercial/career hunters” who actively engaged in hunting for profit, using sophisticated equipment such as guns. The second category were the “farmer/security hunters”, who were essentially farmers or community security men, passively hunting by setting traps around farms and living houses. Generally, we found that the hunter node could relate directly with all other nodes, according to the following excerpts drawn from the KII:

“…*sometimes I supply directly to the consumer, or wholesaler. It depends on the quantity of the catch and sometimes on the price they offer”* (Hunter, 53 yrs. male).

*“We get meat anywhere it will be good and profitable for us, whether wholesaler or hunter, anyone that supply for good price, we buy from them”* (Retailer, 44 yrs. female).

**Fig. 1:**
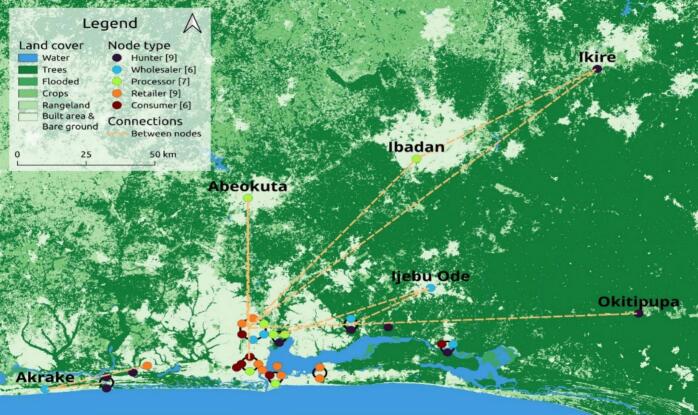
Spatial Distribution of Nodes, Routes and Sources of Wildmeat in-flow of the Lagos Urban Wildmeat Value Chain.

As situations may demand, the hunter node may also have a sub-node: “hunter-processor” to carry out a first-stage processing such as evisceration and cutting into parts for easy transportation from the forest/hunting zone. Also, it is observed that the processor node is the second stage in the chain, with the processors' main activity being to prepare the meat for further distribution or consumption. There is also existence of a sub-node here: the “retailer-processor”, a merging of both roles for profit maximization. The spatial distribution of the nodes in the value chain is shown in Fig. 1**.**

**Fig. 2:**
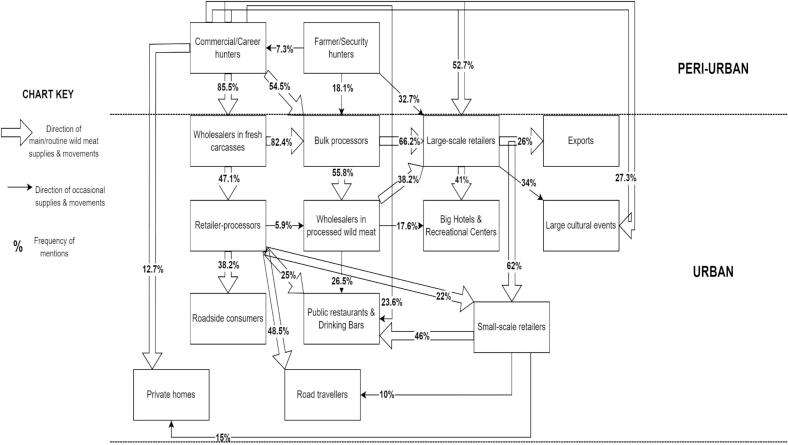
Flow of wild meats and actors' interactions within the wild meat value chain

While the hunter nodes were located along the internal borders of Lagos city (Epe, Ikorodu and Badagry), the consumer nodes were clustered at the Surulere, Eti-Osa, and Lagos Island axis in the Lagos metropolis. The wholesaler nodes were co-located with the hunter nodes, and the processor and retail nodes were spread round, depicting more value chain activities at these nodes. Wild meat products in fresh and processed forms were also directly received into Lagos from five external sources within Nigeria. These were in Abeokuta, Ibadan (processor nodes), Ikire, Okitipupa (hunter nodes) and Ijebu-ode (wholesaler node). An international/external wild meat source node was seen: Akrake, a border town in the Republic of Benin lying in close proximity to Nigeria. The flow and movement of wild meat products within the value chain is shown in Fig. 2**.**

The wholesaler node was seen operating as intermediary between the hunter and processor, processor and retailer or even hunter and retailer. This node was typically a middleman business stage of the supply chain, receiving the majority (85.5 %) of fresh carcasses from commercial hunters. The following excerpts show the hunter-wholesaler-processor– retailer relationship:

*“I buy in bulk from hunters and give to a processor whom I pay to process for me, and I then sell to restaurants and hotels”* (Wholesaler, 45 yrs. Male).

*“I have many hunters that hunt for me. I go to the sites where the hunters usually gather after hunting from the forests. That is how I get [the carcasses] and transport to places where other traders are also waiting to buy from me.”* (Wholesaler, 57 yrs., Male).

*“Who I buy from depends on availability, but majorly I buy processed meat from processors. But sometimes, we are lucky to find wholesalers who sale directly to us”* (Retailer, 41 yrs. female).

*“My main source of supply is the hunters, but sometimes I buy processed meat from processors who also buy from hunters”* (Wholesaler, 39 yrs. Male).

After the wholesaler node, the processor node was next. They were divided into bulk processors and retailer-processors. While the bulk processors supplied wild meat to wholesalers of processed products (58.8 %) and retailers (62.2 %), retailer-processors supplied to road travelers (48 %), public restaurants and drinking bars (25 %), and small retailers (22 %).

The retailer node was next. This node had actors playing sub-roles such as large-scale retailers and small-scale retailers. Large-scale retailers said they supplied wild meat products to majorly small-scale retailers (62 %), big hotels and recreational centers (41 %), large cultural events (34 %), and for exports (26 %). It was the node that had routine direct contact with consumers; although some hunters also had this direct contact in cases where a consumer buys directly from the hunter. While actors at other nodes said they also consume wild meat, no distinct consumer agreed to participate in the study, citing fear associated with personal perceptions of publicity and stigmatization. However, participants' responses from the other nodes gave insights on the consumer as the distal and final node of the wild meat value chain.

### 3.2 Temporal characteristics of the value chain

**Fig. 3:**
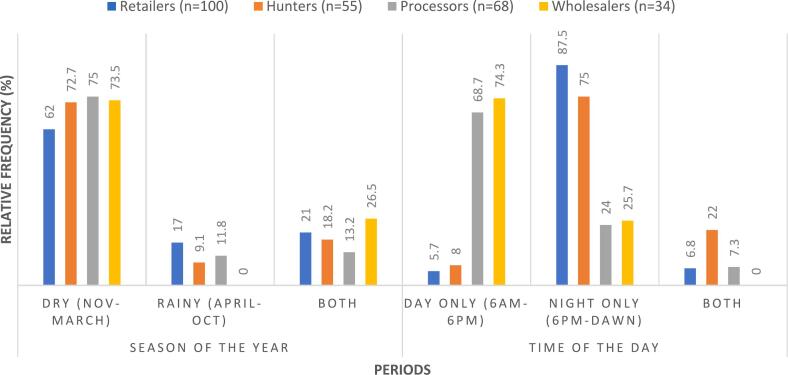
Temporal variations in node activities in the Lagos Wildmeat Value Chain.

There were varying times and seasons of optimum activity in the value chain (Fig. 3). All actors were most active during the dry season (≥ 62 %), followed by both seasons (≤ 26.5 %), and were least active during the rainy season (≤ 17 %). Only 19 % of actors were busy all year round. Also, results showed diurnal/nocturnal differences in the operations of the nodes. While hunters (75 %) and retailers (87.5 %) preferred to operate only at night, wholesalers (73.5 %) and processors (68.7 %) operated during the day only. Only 9 % of all actors reported activities during both day and night times.

### 3.3 Characterizing the actors by gender, age and motivation

Table 1 shows data on the socio-demographic characteristics of the 257 respondents to our questionnaires, revealing a diverse representation across various demographic categories.

**Table 1:** Data on actors' gender, age and motivation.

ACTOR CATEGORY	GENDER		AGE		MOTIVATION	
	Male (%)	Female (%)	Age Range	Frequency (%)	Motivation	Frequency (%)
RETAILERS (n = 100)	17.0	83.0	≤18	2 **(2.0)**	Income	97 **(97.0)**
			18–40	38 **(38.0)**	Food	3 **(3.0)**
			41–55	45 **(45.0)**	Hobby	**0 (0)**
			≥55	15 **(15.0)**	Family tradition	0 **(0)**
HUNTERS (n = 55)	100	0	≤18	–	Income	41 **(74.6)**
			18–40	18 **(32.7)**	Food	8 **(14.5)**
			41–55	31 **(56.4)**	Hobby	2 **(3.6)**
			≥55	6 **(10.9)**	Family tradition	4 **(7.3)**
PROCESSORS (n = 68)	10.3	89.7	≤18	3 **(4.4)**	Income	55 **(80.9)**
			18–40	6 **(8.8)**	Food	13 **(19.1)**
			41–55	41 **(60.3)**	Hobby	0 **(0)**
			≥55	18 **(26.4)**	Family tradition	0 **(0)**
WHOLESALERS (n = 34)	38.3	61.7	≤18	–	Income	100 **(100)**
			18–40	6 **(17.6)**	Food	0 **(0)**
			41–55	20 **(58.8)**	Hobby	0 **(0)**
			≥55	8 **(23.5)**	Family tradition	0 **(0)**

The gender distribution revealed that although males accounted for 100 % of the hunter node, female actors generally outnumbered males in the value chain, constituting 64.2 % of the total sample population. The processor and retail nodes specifically were female-dominated (86.7 % and 83 % females respectively). The predominant age range of actors was 40–55 years (53.3 %), followed by 18–40 (26.5 %). Only a few participants were below the age of 18 or above the age of 55 (18.3 % and 1.9 % respectively). In exploring their motivations, a clear pattern emerged. The overwhelming majority (88.3 %) engaged primarily for income generation, while smaller percentages cited motivations of food (9.3 %), family tradition (1.6 %) and hobby (0.8 %).

Retailers (*n* = 100) exhibited a diverse age range, with 45 % falling between 40 and 55 years of age. The great majority (97 %) were motivated by income, emphasizing the economic aspect of their involvement. None of the retailers reported engagement for hobby or other reasons, highlighting the overwhelmingly financial nature of their participation. On the other hand, hunters (*n* = 55) showed a varied age distribution, with a substantial percentage (56.4 %) falling within the 40–55 age group. Their primary motivation was income (74.6 %), underscoring the economic reliance on wild meat activities. Additionally, some hunters engaged for food (14.5 %) and hobby (3.6 %), revealing a multifaceted motivation profile within this category. For processors (*n* = 68), 60.3 % of actors fell within the age range of 40–55 years (median age = 47.5). Similar to the retailers, income was the predominant motivation for processors (80.9 %), and reported the highest motivation for food (19.1 %). Other motivations were not reported, indicating a focus on practical and sustenance-related incentives. The wholesalers (*n* = 34) were also primarily concentrated in the 40–55 age group (median age = 47.5), and their sole motivation was income (100 %), demonstrating a clear economic drive in their engagements.

### 3.4 Species traded in the lagos wild meat chain

A total of thirty-five (35) species are reported in this study as being traded in the Lagos wild meat value chain. Of these, twenty-five (25) species were mentioned by the participants, while ten (10) additional species were identified by the researchers at the hunter and wholesaler nodes during the field work process.

Table 2 shows a breakdown of all the traded species. These were: rodents (7), ungulates (6), birds (3), primates (3), testudine (1), snakes (6), lagomorph (1), lizards (3), scaly mammals (2), and viverrids (3). Duikers (small to medium-sized ungulates) and grasscutters (large rodents) received the highest mentions (100 % each), followed by porcupines (94.9 %), giant-pouched rats (90.7 %), monitor lizards (84 %), rock pythons (82.8 %), tree hyraxes (80.5 %), pangolins (78.9 %), African civets (75.5 %), monkeys (such as mona monkey and common patas monkey [71.6 %]), sitatunga (69.6 %), and puff adders (59.9 %). The rest were mentioned by less than 50 % of the respondents, which suggest they were less common. These included the pottos (2.7 %), tortoises (6.2 %), African buffalos (6.2 %), and African house snakes (7.0 %). Some species were associated with social norms in the value chain, driven by beliefs rooted in ancient African culture and religion. While some species were treated with respect during hunting (eg sitatunga), some were hunted for their perceived spiritual and medicinal value (pythons), or avoided altogether by some actors due to religious reasons (eg. wild pigs and shrews). Certain animal body parts were not to be sold as meat within the value chain, as they were highly priced as raw materials for zootherapy (eg. heads of monkeys and snakes). This can be seen in the following excerpts:

**Table 2:** Species Traded in the Lagos Wildmeat Value Chain, Frequency of Mentions and their Conservation Statuses.

No.	Common Name (s)	Scientific Name(s)	Local Name(s)	Species Category	Frequency of mentions (%)	Conservation Status
1.	Grasscutter; Cane Rat	*Thryonomys swinderanus; Thryonomys gregorianus*	*Oya*	Rodent	100	Least Concern (IUCN, 2016)
2.	Ogilby's duiker	*Cephalophus ogilbyi*	*Agbonrin; Etu*	Ungulate	100	Least Concern (IUCN, 2016)
3.	Blue duiker	*Philantomba monticola*	*Agbonrin, Etu*	Ungulate	⁎	Least Concern (IUCN, 2016)
4.	African Brush-tailed Porcupine	*Atherurus africanus*	*Oore*	Rodent	94.9	Least concern (IUCN, 2016)
5.	African Giant-pouched Rat	*Cricetomys emini*	*Okete*	Rodent	90.7	Least concern (IUCN, 2016)
6.	Nile Monitor	*Varanus niloticus*	*Antar*	Lizard	84.0	Least concern (IUCN, 2019)
7.	Ball Python	*Python regius*	*Ejonla; Olufa*	Snake	⁎	Near Threatened (IUCN, 2020)
8.	Central African Rock Python	*Python sebae*	*Ejonla; Olufa*	Snake	82.8	Near Threatened (IUCN, 2019)
9.	Western Tree Hyrax	*Dendrohyrax dorsalis*	*Ofafa; awawa*	Rodent	80.5	Least Concern (IUCN 2014)
10.	African Black-bellied Pangolin	*Phataginus tetradactyla*	*Akika*	Scaly mammal	⁎	Vulnerable (IUCN, 2019)
11.	White-bellied Pangolin	*Phataginus tricuspis*	*Akika*	Scaly mammal	78.9	Endangered (IUCN, 2019)
12.	African Civet	*Civettictis civetta*	*Eta; Jakumo*	Vivverid	75.5	Least Concern (IUCN, 2015)
13.	African Palm Civet	*Nandinia binotata*	*Eta; Jakumo*	Vivverid	⁎	Least Concern (IUCN, 2015)
14.	Mona Monkey	*Cercopithecus mona*	*Obo; Ologede*	Primate	71.6	Near Threatened (IUCN, 2019)
15.	Common Patas Monkey	*Erythrocebus patas*	*Obo; Ologede*	Primate	⁎	Near Threatened (IUCN, 2019)
16.	Sitatunga	*Tragelaphus spekii*	*Igala*	Ungulate	69.6	Least Concern (IUCN, 2016)
17.	Puff Adder	*Bitis arietans*	*Mana-mana*	Snake	59.9	Least Concern (IUCN, 2014)
18.	Carpet Viper	*Echis ocellatus*	*Paramole*	Snake	⁎	
19.	Dwarf Crocodile	*Osteolaemus tetraspis*	*Alegba; oni*	Lizard	42.0	Vulnerable (IUCN, 1996)
20.	West African Crocodile	*Crocodylus suchus*	*Alegba; oni*	Lizard	⁎	Not assessed
21.	Striped Ground Squirrel	*Xerus erythropus*	*Okere*	Rodent	42.0	Least Concern (IUCN, 2016).
22.	Thomas's Rope Squirrel	*Funisciurus anerythrus*	*Okere*	Rodent	⁎	Least Concern (IUCN, 2016).
23.	Red River Hog	*Potamochoerus porcus*	*Tuuku*	Ungulate	39.3	Least Concern (IUCN, 2016).
24.	Ahanta Spurfowl	*Pternistis ahantensis*	*Akparo*	Bird	35.4	Least Concern (IUCN, 2016).
25.	Nigerian Shrew	*Crocidura nigeriae*	*Asin; Ekute*	Rodent	33.1	Least Concern (IUCN, 2016).
26.	Rusty-spotted Genet	*Genetta maculata*	*Ogbo-oloko*	Vivverid	29.9	Least Concern (IUCN, 2015).
27.	Forest cobra	*Naja melanoleuca*	*Oka*	Snake	24.9	Least Concern (IUCN, 2019).
28.	African Savannah Hare	*Lepus victoriae*	*Ehoro*	Lagomorph	24.5	Least Concern (IUCN, 2008)
29.	African Blue Quail	*Synoicus adansonii*	*Akparo*	Bird	13.6	Least Concern (IUCN, 2016).
30.	Common African House Snake	*Boaedon fuliginosus*	*Ejo*	Snake	7.0	Least Concern (IUCN, 2014)
31.	African Buffalo	*Syncerus caffer*	*Efon*	Ungulate	6.2	Near Threatened (IUCN, 2018)
32.	West African Black Forest Turtle	*Pelusios niger*	*Ijapa*	Testudine	6.2	Near Threatened (IUCN, 2018)
33.	Crested Guineafowl	*Guttera verreauxi*	*Awo*	Bird	⁎	Least Concern (IUCN,
34.	West African Potto	*Perodicticus potto*	*Ikin*	Primate	2.7	Near Threatened (IUCN, 2020)
35.	Black duiker	*Cephaolophus niger*	*Etu; Agbonrin*	Ungulate	⁎	Least Concern (IUCN, 2016)

⁎ *Species not mentioned by respondents but identified by the researchers during participants' observation.*

*“Never you hunt a deer [sitatunga] when your wife pregnant. If you see it in the forest, it may walk [up] to you. If you shoot [at] it, then you have killed your child”* (Hunter, 66 yrs. Male).

*“Look very well at the snakes here…you will not see their heads. Immediately after hunting, hunters cut off the snake heads for sale to people who use it for traditional medicines”* (Wholesaler, 43 yrs. Male).

“*As a Muslim, I don't handle wild pigs because [I believe] they are unclean.”* (Hunter, 54 yrs. Male).

### 3.5 Social norms and governance among actors in the value chain

Throughout the value chain, the governance showed a mix of formality and an ingrained informal structure sustained and kept together through trust, rituals and ancient traditional belief systems. Two types of governance structures were identified: (i) a merged general structure comprising all actors, and (ii) a distinct structure comprising wholesalers, processors and retailers.

**Fig. 4:**
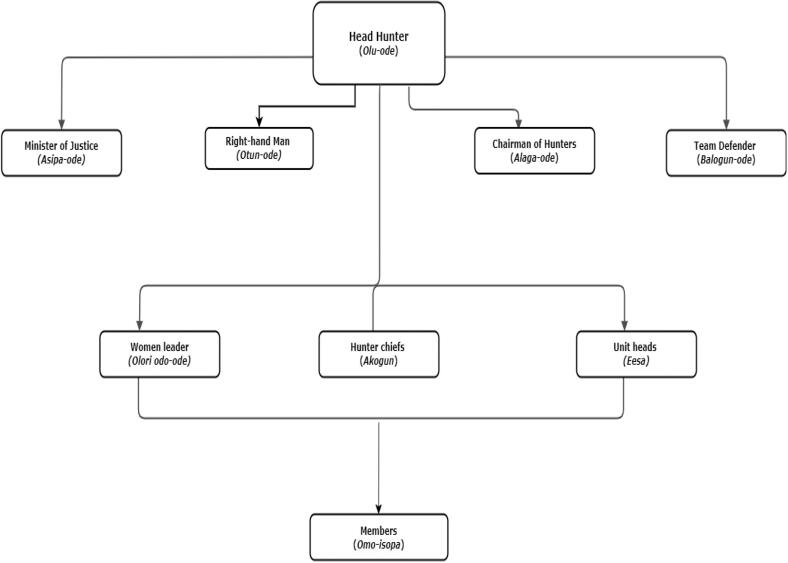
The merged general governance structure among actors in the Lagos wild meat value chain.

In the merged general structure, a head hunter (*Olu-ode*) is responsible for the overall leadership within the group (Fig. 4). He works with a team of leaders to ensure cohesion and progress of the group. The “*Otun-ode*” is the head hunter's trusted ally, loyalist and right-hand man who plays an advisory role. The *Asipa-ode (minister of justice*) mediates the resolution of conflicts among members. To achieve this, he employs different options such as dialogues and the casting of lots among concerned persons, using kolanuts. In other cases, members themselves adopt traditional informal regulations, according to the following excerpts:

*“During group hunting, if more than one person chased and shot down a particular animal, it is the person who first shot the animal that owns it. That is how we avoid problems with one another”* (Hunter, 58 yrs. Male).

*“After group hunting, when we all gather to share the meat, the head is usually given to the person who owns the animal. Then we divide all other body parts equally” (Hunter, 43* *yrs Male).*

If the disputes cannot be resolved by dialogue, casting of lots and regulations, the *Asipa-ode* resorts to traditional spiritual incantation and divination, as captured in an excerpt:

*“We have superior means of solving difficult conflicts. Our Asipa-ode can either consult the spirits of our dead legend hunters for answers, or ogun, the god of hunters who delivers justice” (Hunter, 64* *yrs Male).*

The team defender/war lord (*Balogun-ode*) was said to be responsible for protecting the members and territory from external attacks, using several skills which may include combat and spiritual enchantments as found suitable. The chairman of hunters (*Alaga-ode*) directly supervises the chiefs (*Akogun*) and unit heads (*Eesa*), who work hand-in-hand to ensure discipline and strict adherence of members (*Omo isopa*) to their norms and practices. The women leader (*Olori odo-ode*) collects levies and also attends to welfare and gender concerns. Notwithstanding the presence of women in the informal governance structure of the hunters, it is interesting to note that all the hunter respondents in the study were male. The answers from the KII participants portrays a continual attachment of the hunters to their ancient traditions which also showed gender aspects, as is seen in the following excerpts:

*“… yes, the women are part of the governance because they have a part to play with the gods of the hunting process. I can't say more than that, please.”* (Hunter, 45 yrs. Male).

*“Our tradition does not overlook the position of the women. They are part of the hunting support system. But a woman must not join in hunting, or lead a hunting group”* (Hunter, 41 yrs. Male).

Processing of wild carcasses was regarded as the sole responsibility of women, with men having other responsibilities that depict physical strength and rigors, such as hunting. If a man engaged in processing, then he was likely to face societal stigmatization, as seen in the excerpt below:

*“Here, cutting into the belly and cleaning the intestines as well as cooking it is the work of women. A man should never be seen doing that. If he does, then he is [considered] a weakling”* (Wholesaler, 52 yrs. Male).

**Fig. 5:**
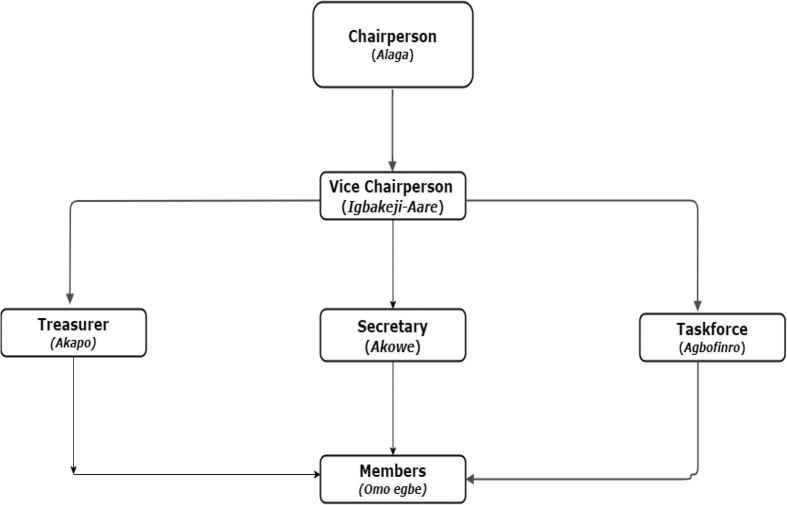
The distinct wholesaler/processor/retailer governance structure of the Lagos wild meat value chain.

In the wholesaler/processor/retailer system, the governance structure was anchored on five main leaders: the chairperson (*Olori-ogbe*), vice-chairperson (*Igbakeji-Aare*), secretary (*Akowe*), treasurer (*Akapo*) and a head of taskforce (*Agbofinro*) (Fig. 5). A similar structure was evident in the sub-groups at the different wild meat markets, sales outlets and aggregation points. In this structure (which is dominated by females), members pay levies and other financial contributions for needy members' welfare. Another example of such levies was the waste management levy either imposed by the government or local market authorities. Although not written on paper, there were regulations or codes-of-conduct guiding members' activities. These included informal codes such as “no befriending of a colleague's spouse” and “having no dealings or business with a colleague's buyer or supplier”. Defaulters face penalties, as seen in the following interview excerpts:

*“Yes, there are some regulations. Although not written, we discuss and we keep [to] it. If you don't, [the] taskforce will ask for fine or send you [on] suspension, depending [on offense]” (Retailer, 38* *yrs Female).*

*“….as a member you should not befriend or take another's wife or husband. Also, as a member you should not do business with another's supplier or customer….., [if you do] we will expel you immediately” (Processor, 57* *yrs Female).*

Penalties for defaulters included monetary fines, suspension of activities, or outright expulsion. As seen from the excerpt above, punishments were dependent on the severity of offense, and often enforced or communicated to the offender by the head of taskforce. There were also fines enforced on actors who defaulted on cleaning their premises (drinking bar, restaurant or market stall). This was enforced by the local government through the ministry of environment. These structures and mechanisms ensure network cohesion and progression of activities within the value chain.

## 4 Discussion

Our study gives a better understanding of the wild meat value chain. Lagos is the largest urban center in Africa, well connected to the world and as this study shows has a thriving wild meat trade that draws wild meat from across Nigeria and a neighboring West African country (Republic of Benin). This portends varying implications for global health, biodiversity conservation, trade regulation, and gender development.

### 4.1 Potential public health risks of the value chain

Our study found that women were more exposed to potential risks of zoonotic pathogens through their predominant role of wild meat processing. This was also reported in a study [28] which found that women were twice more likely to process wild meat than men, signifying a gendered difference in zoonotic pathogen exposure risks in the value chain. Interestingly too, study results revealed that hunters and processors consumed part of their bushmeat, probably out of convenience and accessibility. This poses hygiene risks, especially for hunters since they are unlikely to maintain sanitary measures while butchering wild meat in the forests. Also, despite the presence of intermediaries (wholesalers), we found that actors in the hunter nodes could relate directly with other nodes in the value chain. This denotes the hunter node as a critical node for disease control, as any zoonotic risks associated with this node can directly impact the other nodes. The increase in wild meat processing’ activities during the dry season could be corelated to increased hunting activities at this period. This indicates that the dry season is the period at which the risk of zoonotic spillover in the value chain is greatest. Although 9 % of actors were engaged all year round, no wholesaler reported being busiest during the rainy season (0 %). While our study results do not clearly establish a link between this finding and wild meat demand, we posit that the poor visibility, access and hunting weather conditions associated with the season could be a determinant factor. This suggests challenges or limitations in the wild meat supply chain during the rainy seasons. Inversely, this could play a positive role to aid ecosystem resilience and replacement of wildlife deficits through breeding during this season. Our results showed that the distal nodes (processor and retailer) received their supplies from the wholesaler node. As also found in a study [29], the wholesaler node played a critical role of connecting supply and demand within the value chain, thus influencing distribution and market access. The SARS-CoV-2 outbreak emphasized that urban markets presented favorable environments for zoonotic pathogens to emerge and spillover [30,31]. Hence, the activity of actors at this node can be said to be significant and a strong determinant factor in the spread of potential zoonotic pathogens into the human population through wild meat. Additionally, species belonging to taxa reported in our study (eg. pangolins, monkeys, reptiles, small carnivores, rodents, wild pigs, etc) have been globally documented as common reservoirs of zoonotic pathogens [9,[32], [33], [34]]. For example, monkeys, also reported in this study, have been linked to the transmission of Monkeypox virus to humans, which led to different outbreaks [35]. With 43 % of emerging infectious diseases originating from wildlife [36], the hunting, handling, trade or consumption of these reservoir species further puts the actors at higher risk of contracting zoonotic diseases.

### 4.2 Characteristics of the value chain

The hunter nodes represented the beginning of the value chain, making them the source node. While networking and trade in processed wild meat products took place within the Lagos metropolis, substantial supplies of fresh carcasses came from suburbs outside the city. Hence, actors at the hunter node represented a peri-urban population interfacing with the urban areas through the supply of wild meat products. Our results showed that all wild meat supplies came from these areas.

The identification of hunter nodes as the primary source of wild meat in this study (rather than from wildlife farming, or from opportunistic off-take such as from road-kills or fence entanglements) underscores the central role that hunters play in the extraction and supply of wild animals for consumption, and highlights the need to address sustainable hunting practices and regulate the trade at its source. This finding aligns with previous research by Koppert et al. [29] who estimated that approximately 30 %–80 % of wild meat consumed in Central Africa was obtained through hunting. Additionally, though belief-based motivation (family tradition) accounted for only 7.3 %, interview responses showed that actors at the hunter node were predominantly practitioners of the traditional African culture as practiced by the “*yorubas”,* the ethnic group and language of the people of Southwestern Nigeria [37,38]. Hence, there was an intricate web of beliefs and societal norms intertwined with specific wildlife species and hunting in general. Also, substantial evidence from our thematic analysis of social norms and practices evident at the general governance system led by hunters suggests that social norms and practices of the actors could be an important factor sustaining wild meat trade in Lagos.

The intermediary role of wholesaler nodes in facilitating the movement of wild meat from forest hunting areas to urban markets in Lagos highlight their significance in shaping market dynamics and trade patterns. The processor node represents the value addition stage of the commodity chain, where wild carcasses are transformed from their raw states to visually appealing and eatable products through cooking, drying, roasting, and other processes. By prioritizing consumers' wild meat preferences, the retailer node played a crucial role in shaping consumer behavior and market demand. Although mostly motivated by income (97 %), the role of the retailer node in the chain underscores its significance in regulating potential public health risks associated with wild meat. Compared to the hunter, wholesaler and processor nodes, the retailer node is better positioned for consumer protection by ensuring the safety and quality of wild meat. The interconnected relationships between nodes, such as direct supply from commercial hunters to consumers (totaling 40 % responses), highlight the complexity and dynamics of the wild meat trade network. This also resonates with the complex network structure of wildlife trade routes quantitatively as previously mapped out by [39].

Furthermore, income emerged as the overarching motivation across all actor categories (88.1 %), emphasizing the economic significance of urban wild meat value chains. Food (9.2 %) and hobby motivations (0.9 %) were minimal and very low across all categories, indicating a prevailing focus on the economic aspects rather than recreational engagement. These align with findings of a quantitative survey [16] which found income motivations as a major driver of wild meat trade activities in Africa.

Although five main nodes were identified along the value chain, this study examined four nodes and excluded the consumer node. This was due to challenges experienced during data collection: the lack of consent of identified consumers in responding to questions or filling of the structured questionnaires; and the overlapping nature of the consumer node with the other nodes. Also, though sub-categories were identified within the main actor groups (eg. farmer/security hunters, retailer-processors, etc), it was difficult to study them independently because they frequently switched their roles. Hence for our quantitative analysis, they were grouped as hunters, wholesalers, processors, and retailers. Furthermore, our study results showed inflow of wild meat from the Republic of Benin. However, we did not quantify the inflow or establish if such internationally-sourced meat was intended for use within the metropolis, or for exportation through the Lagos trade corridor. This aspect was beyond the scope of this study.

### 4.3 Regulations and gender development

The inclusion of women in the informal traditional governance structures within the hunter node as observed in our study, reflects an important aspect overlooked in other similar studies [2,9,40]. It points to the diverse and significant roles that women play, especially highlighting gender inequality in roles in the Lagos wild meat value chain. Government sanitary enforcement was evident at the women-dominated processor and retailer groups, suggesting a probable external influence on the value chain. It was also seen that the merged wholesaler/processor/retailer groups assumed a more formal governance structure; suggesting that when operating independent of the male-dominated hunters' group, the female value chain actors ran a system which could be more effectively monitored and regulated by the government. This finding contributes evidence-based data which could be useful in the designing of gender-friendly interventions to discourage wild meat trade and also reduce the potential negative impacts of the occupation on women.

### 4.4 Wildlife ecology and conservation

The identification of a diverse range of wildlife species highlights the global biodiversity implications of the wildlife trade, which was also emphasized by Reino et al. [41]. We report duikers and grasscutters as the commonly hunted species in our study. This also corroborates the findings of Phelps et al. [42] who quantitatively demonstrated the popularity of these species in the wildlife trade due to factors such as their abundance accessibility and cultural preferences for their meat. This alignment underscores the consistency of species preferences across different regions and provides empirical support for the observed patterns in our study. Moreover, our study's recognition of less common species such as potto, buffalo and turtle, provides a clearer insight of the wild meat market diversity. While certain species may have dominated the trade due to their popularity, the presence of less common species signify the role of species abundance, ease of capture and cultural norms in modulating species availability in wild meat markets. For example, our study results suggest that social norms such as cultural taboos and religious restrictions on some species (eg. red river hog and Nigerian shrew) may be a factor affecting their frequency.

Furthermore, quantitative assessments of species' ecological traits and habitats are crucial for evaluating the potential ecological consequences of trade activities. Hence, the involvement of species from different ecological niches as seen in our study emphasizes the ecological impact of wildlife trade, as noted by Reino et al. [41]. The exploitation of species from diverse ecological niches may have cascading effects on ecosystems, including changes in species interactions, habitat degradation, and loss of biodiversity [43,44]. Our study's recognition of some species as less common in the market may dis-align with research highlighting variations in species popularity and market demand, as noted by Ingram et al. [17]. Their quantitative analysis of market dynamics revealed fluctuations in species popularity and demand driven by factors such as cultural preferences, economic trends, and regulatory measures [17]. Therefore, while certain species may be less common in the market at a particular time, their popularity and demand may vary over time and across different regions. Understanding these variations are essential for adaptive conservation strategies that respond to changing market dynamics and prioritize species at risk of overexploitation. The majority of species reported in our study are listed under the “least concern” category by the International Union for Conservation of Nature red list of threatened species [45]/ Despite this, a few examples of species listed as vulnerable are being traded, such as the dwarf crocodile which has been listed as “vulnerable” since 1996. Since the conservation status assessments of the majority of species reported in our study were conducted within the years 1996 to 2016, it could therefore infer that the listings are based on old data which do not reflect the current statuses of these species. Therefore, there is a need for their immediate re-assessment to inform adequate conservation measures.

## 5 Conclusion

Key findings from this study highlight the pivotal role of hunter nodes as the primary source of wild meat, the interconnected relationships between different nodes, the mixed informal/formal governance structures, and the diversity of wildlife species involved in the value chain. The increase in wild meat activities in the dry season points to a period of increased zoonotic transmission risks, hence should serve as entry point for public health interventions. Such interventions should prioritize the nodes with higher risks of exposure to zoonotic pathogens (hunters, wholesalers and processors). The recognition of women's roles in wild meat harvesting traditions and the continuity of traditional practices underscore the importance of cultural and gender determinants in wild meat value chains [46]. Empowering women in wild meat value chains and incorporating their perspectives into conservation and public health policies can lead to a more effective and sustainable outcomes. Hence, conservation efforts and zoonotic risk prevention strategies should be gender-inclusive. Lastly, conservation efforts should prioritize frequently-traded species, and vulnerable communities should be provided alternative livelihood initiatives to lessen wild meat hunting while meeting socioeconomic demands. Such initiatives may include co-management of forest resources (eg engaging community members as rangers), provision of agribusiness start-up funding support, and ensuring cheaper access to meat from farmed animals.

## CRediT authorship contribution statement

**Samuel N. Akpan:** Writing – original draft, Methodology, Investigation, Formal analysis, Data curation. **Pim van Hooft:** Writing – review & editing, Supervision. **Anise N. Happi:** Writing – review & editing, Validation, Project administration. **Ralph Buij:** Writing – review & editing, Validation, Supervision, Funding acquisition, Conceptualization. **Frank van Langevelde:** Writing – review & editing, Supervision, Resources, Funding acquisition. **Elizabeth A.J. Cook:** Writing – review & editing, Supervision. **James M. Hassell:** Writing – review & editing, Visualization, Project administration, Conceptualization. **Dawn M. Zimmerman:** Writing – review & editing, Funding acquisition. **Sherrill P. Masudi:** Visualization, Software. **Christian T. Happi:** Supervision, Resources, Funding acquisition. **Lian F. Thomas:** Writing – review & editing, Visualization, Supervision, Methodology, Formal analysis.

## Declaration of competing interest

The authors declare that they have no known competing financial interests or personal relationships that could have appeared to influence the work reported in this paper.

## Data Availability

Data will be made available on request.

## Appendix A Supplementary data

Supplementary material 1 Image 1

Supplementary material 2 Image 2

Supplementary material 3 Image 3

Supplementary material 4 Image 4

Supplementary material 5 Image 5
