# Supplementary material for: Structure, conservation and health implications of urban wild meat value chains: A case study of Lagos, Nigeria
Source: One Health. 2025 Feb 14;20:100992. doi: 10.1016/j.onehlt.2025.100992 (PMC11876908; doi:10.1016/j.onehlt.2025.100992)
Supplement: Supplementary file 2 — Supplementary material 2 [file mmc2.docx]

**QUESTIONNAIRE**

**Study Title:** Investigating the structure of the Lagos wild meat value chain

**Principal Investigators:** Anise Happi & Christian Happi.

**Field Coordinator:** Samuel Akpan (PhD student)

**Questionnaire ID:**

**Date:**

**Area:**

Do you consent to participate in this study? Yes No

** Kindly tick the option(s) as it applies to you.*

1. **Demographics of the participant**

*(This section is for your basic personal information. This information will help us in better understanding of actors in the value chain).*

1. What is your age?
2. ≤18 years
3. 18-40 years
4. 40-55 years
5. ≥55 years
6. What is your gender?
7. Female
8. Male
9. What is your level of education?
10. Primary
11. Secondary
12. College
13. University
14. Informal education
15. None
16. What is your religion?
17. Muslim
18. Christian
19. Traditionalist
20. None
21. **Value Chain Structure**

*(This section seeks to assess your role in the wild meat value chain, other actors involved, species traded, the temporal and spatial characteristics of the value chain).*

1. What is your role in the value chain?
2. Hunter
3. Wholesaler
4. Processor
5. Retailer
6. Consumer
7. For what purposes do you trade in wildmeat?
8. For income
9. For food
10. For religious purpose
11. For medicinal purpose
12. Other reasons (please specify)
13. Which animal(s) species do you trade?
14. How often do you trade wildmeat?
15. every day
16. every week
17. every month
18. Occasionally
19. I don´t know
20. Which season is the busiest for your activities?
21. Rainy season
22. Dry season
23. All seasons
24. None
25. What time of the day is your business mostly active?
26. In the Day time (6pm-6am)
27. At night (6pm-6am)
28. At all times (both night & day)
29. From whom do you obtain the wildmeat?
30. I hunt it myself
31. Hunters
32. Wholesalers
33. Processors
34. Retailers__________________________________________________
35. To whom do you supply/sell your products?

a) Hunters

b) Wholesalers

c) Processors

d) Retailers

e) Consumers (please specify)

1. From which location do you obtain the wildmeat?
2. Within Lagos
3. Outside Lagos (please specify)
4. I do not know
5. Do you process wildmeat in any way?
6. Yes
7. No
8. If yes to the question above, how do you process the wildmeat?
9. Skinning
10. Evisceration
11. Salting
12. Smoking
13. Grilling
14. Boiling/cooking
15. Other (please specify)
