# Supplementary material for: Structure, conservation and health implications of urban wild meat value chains: A case study of Lagos, Nigeria
Source: One Health. 2025 Feb 14;20:100992. doi: 10.1016/j.onehlt.2025.100992 (PMC11876908; doi:10.1016/j.onehlt.2025.100992)
Supplement: Supplementary file 3 — Supplementary material 3 [file mmc3.docx]

**KEY INFORMANTS’ INTERVIEW GUIDE**

(Duration: 25-40 minutes)

What is/are your role(s) in the value chain?

From where/whom do you obtain the wild meat?

In what state do you obtain the wild meat (fresh whole carcass, processed, etc)

To whom do you supply your products?

Are there any specific practices you engage in?

Is there a group or association for wildmeat hunters or sellers in your area/market/community? If yes, are you a member?

Who are the leaders? (Kindly describe the leadership structure of the group)

How do you govern yourselves, and are there any regulations or codes-of-conduct that guide your activities or practices?

If yes, what are the regulations or codes-of-conduct?

Are there any punishments for defaulters?

*Other information (follow leads and probe further………………………………*
