# Supplementary material for: Structure, conservation and health implications of urban wild meat value chains: A case study of Lagos, Nigeria
Source: One Health. 2025 Feb 14;20:100992. doi: 10.1016/j.onehlt.2025.100992 (PMC11876908; doi:10.1016/j.onehlt.2025.100992)
Supplement: Supplementary file 4 — Supplementary material 4 [file mmc4.pdf]

MAIN THEMES

SOCIAL NORMS

GOVERNANCE

ZOOTHERAPY

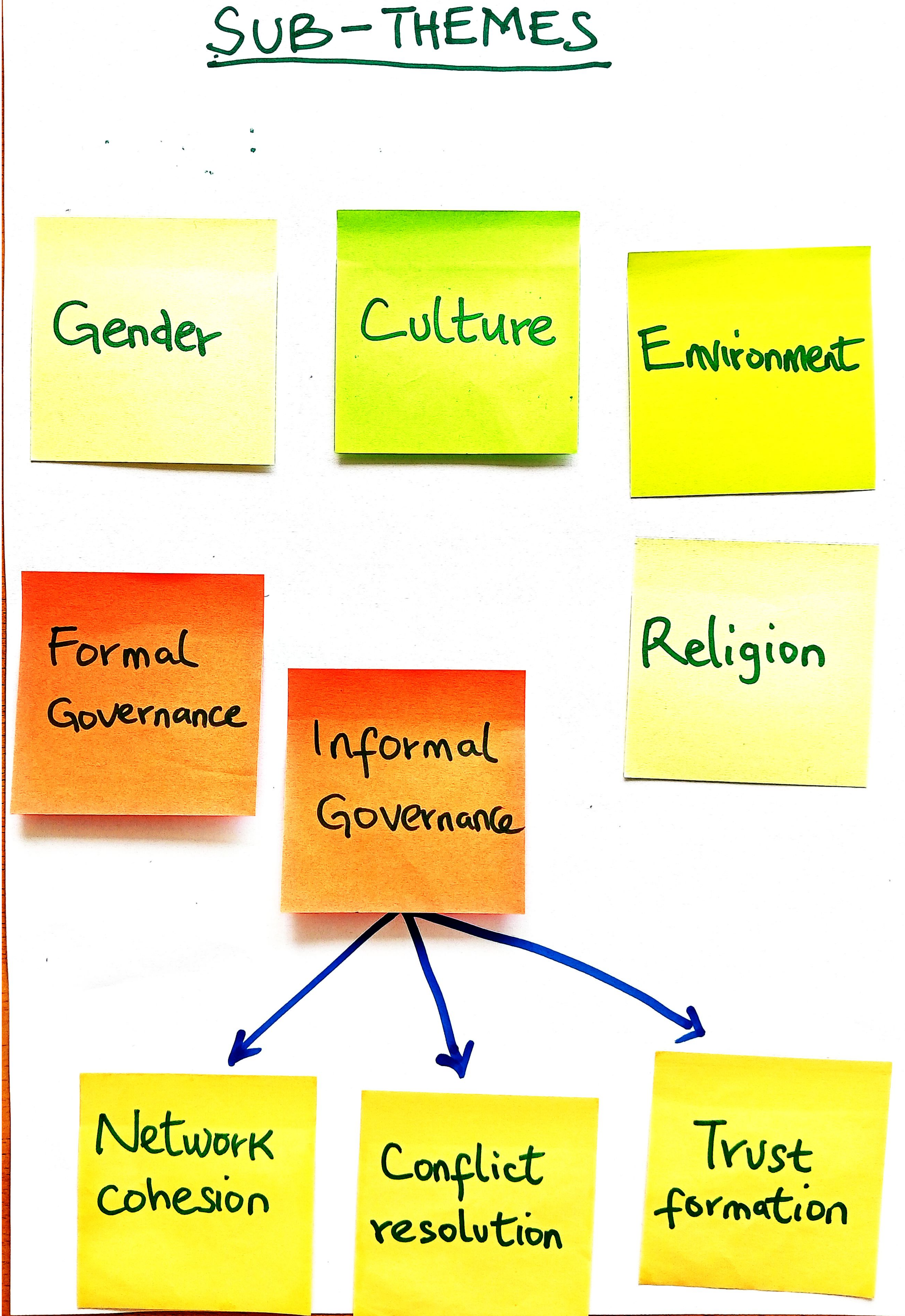
SUB-THEMES
Gender
Culture
Environment
Formal Governance
Informal Governance
Religion
Network cohesion
Conflict resolution
Trust formation

Sub-theme:

GENDER

Women process the carcass

Women dont enter the forests for hunting

A man should not be seen processing carcass

Women have significance with the "gods" of hunting

Sub-theme : Culture

Dividing of python in sixteen portions

Showing of respect to some species like "deer"

Giving head of hunted animal to the owner

Hunting mainly at night

Using of dogs as part of hunting tools

Divination and Incantations

ZOOTHERAPY

Certain animal body parts not sold for meat

Showing of respect to certain species

RELIGION

No hunting during Ramadan fasting

No handling of unclean species

Sub-theme

Formal Governance

Punishments and fines for defaulters

Stick to your customer or suppliers

Sub-theme

Informal Governance

(conflict)
The one first shoots an animal is the owner

Casting of lots
(Team cohesion)

Divination & Incantation
(conflict resolution)

(Team cohesion)
Sticking to one's supplier or buyer

(conflict resolution)
Giving the head to owner of hunted carcass

No befriending another's spouse
(Trust)
